# Characterization of thimet oligopeptidase and neurolysin activities in B16F10-Nex2 tumor cells and their involvement in angiogenesis and tumor growth

**DOI:** 10.1186/1476-4598-6-44

**Published:** 2007-07-09

**Authors:** Thaysa Paschoalin, Adriana K Carmona, Elaine G Rodrigues, Vitor Oliveira, Hugo P Monteiro, Maria A Juliano, Luiz Juliano, Luiz R Travassos

**Affiliations:** 1 Department of Microbiology, Immunology and Parasitology, Experimental Oncology Unit (UNONEX), Federal University of São Paulo, São Paulo, Brazil; 2 Department of Biophysics Federal University of São Paulo, São Paulo, Brazil; 3 Department of Biochemistry, Federal University of São Paulo, São Paulo, Brazil; 4 UNONEX, Department of Microbiology, Immunology and Parasitology (UNIFESP), Rua Botucatu, 862, 8° andar, São Paulo, SP 04023-062, Brazil

## Abstract

**Background:**

Angiogenesis is a fundamental process that allows tumor growth by providing nutrients and oxygen to the tumor cells. Beyond the oxygen diffusion limit from a capillary blood vessel, tumor cells become apoptotic. Angiogenesis results from a balance of pro- and anti-angiogenic stimuli. Endogenous inhibitors regulate enzyme activities that promote angiogenesis. Tumor cells may express pro-angiogenic factors and hydrolytic enzymes but also kinin-degrading oligopeptidases which have been investigated.

**Results:**

Angiogenesis induced by B16F10-Nex2 melanoma cells was studied in a co-culture with HUVEC on Matrigel. A stimulating effect on angiogenesis was observed in the presence of B16F10-Nex2 lysate and plasma membrane. In contrast, the B16F10-Nex2 culture supernatant inhibited angiogenesis in a dose-dependent manner. This effect was abolished by the endo-oligopeptidase inhibitor, JA-2. Thimet oligopeptidase (TOP) and neurolysin activities were then investigated in B16F10-Nex2 melanoma cells aiming at gene sequencing, enzyme distribution and activity, influence on tumor development, substrate specificity, hydrolytic products and susceptibility to inhibitors. Fluorescence resonance energy transfer (FRET) peptides as well as neurotensin and bradykinin were used as substrates. The hydrolytic activities in B16F10-Nex2 culture supernatant were totally inhibited by *o*-phenanthrolin, JA-2 and partially by Pro-Ile. Leupeptin, PMSF, E-64, Z-Pro-Prolinal and captopril failed to inhibit these hydrolytic activities. Genes encoding M3A enzymes in melanoma cells were cloned and sequenced being highly similar to mouse genes. A decreased proliferation of B16F10-Nex2 cells was observed in vitro with specific inhibitors of these oligopeptidases. Active rTOP but not the inactive protein inhibited melanoma cell development in vivo increasing significantly the survival of mice challenged with the tumor cells. On Matrigel, rTOP inhibited the bradykinin – induced angiogenesis. A possible regulation of the homologous tumor enzyme in the perivascular microenvironment is suggested based on the observed rTOP inhibition by an S-nitrosothiol NO donor.

**Conclusion:**

Data show that melanoma cells secrete endo-oligopeptidases which have an important role in tumor proliferation in vitro and in vivo. rTOP inhibited growth of subcutaneously injected B16F10-Nex2 cells in mice. TOP from tumor cells and bradykinin in endothelial cells are two antagonist factors that may control angiogenesis essential for melanoma growth. A regulatory role of NO or S-nitrosothiols is suggested.

## Background

Angiogenesis is a fundamental process in tumor growth, providing nutrients and oxygen to the tumor cells. This complex process involves extensive interplay between cells, soluble factors and ECM components. Among the soluble factors, secreted peptidases by tumor and neighbor cells can have a significant role in both tumor development and angiogenesis. Tumor cells express many different types of proteases that are associated with tumor invasibility [1]. Considering the various specificities of secreted and membrane-bound hydrolytic enzymes in the invasive melanoma a diversity of products can be generated. Peptide fragments can stimulate tumor cells to produce oligo-, amino- and carboxipeptidases for further degradation giving rise either to biologically active peptides (growth factors, regulators or signalling ligands), or to substrates accessible to be used as nitrogen source.

Presently, we describe the stimulating effect of B16F10-Nex2 melanoma cells on endothelial cells in a co-culture model of angiogenesis on Matrigel in vitro. In contrast, an inhibitory effect of melanoma cell culture supernatant was observed. The agents responsible for these effects were investigated.

We detected the expression of oligopeptidases in murine melanoma cells of high invasiveness. The homologous mammalian enzymes of the M3A subfamily are generally found in different tissues and cellular compartments. They are neurolysin (EC 3.4.24.16) [2,3] and thimet oligopeptidase (TOP, EC 3.4.24.15) [4], exhibiting similar substrate specificities and possessing a highly conserved HEFGH metal binding motif [5,6]. They were originally described as having 60% sequence identity, and distribution in the cytosol, endoplasmic reticulum, mitochondria and nucleus of different mammalian tissues and tumor cells [7-9]. Membrane-associated forms of these enzymes have been described in corticotrophic tumor cells [10], neuronal cell lines [11] and neurons [12,13] and the secreted forms in neuronal cell line [14-16] cultures.

Both peptidases are known to hydrolyze in vitro various bioactive peptides, including bradykinin (BK) [17], and numerous reports have linked the enzymes to the metabolism of these peptides in vivo [18-23]. BK, generated through the action of kallikreins on a precursor kininogen substrate, induces inflammation, increased vascular permeability, stimulation of the endothelial isoform of nitric oxide (NO) synthase, and vasodilation. Pathological conditions, such as myocardial ischemia, hypertension and cancer are deeply influenced by the kallikrein/kininogen/kinin system. Evidence suggests that part of the cardioprotective effects of specific inhibitors of the angiotensin I-converting enzyme (ACE) and neutral endopeptidase (NEP) is due to the enhanced BK activity [24,25]. Schriefer et al. [26] demonstrated that inhibition of TOP precludes degradation of endogenous BK and provides long-lasting protection from myocardial ischemia/reperfusion injury. TOP and neurolysin also contribute to BK metabolism in the blood vessels [27].

The BK role on tumor-associated angiogenesis and tumor growth has already been addressed [28]. BK stimulates angiogenesis in a sponge granuloma model, synergistically with interleukin-1 [29]. BK has been implicated in the enhancement of tumor growth via increased permeability of the tumor neo-vasculature [30,31]. Tumor growth and development of tumor-associated angiogenesis are suppressed in kininogen-deficient rats [32,33]. These evidences suggest that BK is a primary mediator of tumor angiogenesis and, consequently, of tumor growth.

In the present work, we have characterized TOP and neurolysin activities in conditioned media, lysate and membrane preparations of B16F10-Nex2 melanoma cells. Furthermore, we used in vivo experiments and in vitro Matrigel angiogenesis assay, to determine the role of oligopeptidases released by B16F10-Nex2 melanoma cells on tumor growth and BK-dependent angiogenesis. We suggest that locally produced NO could play a role in the regulation of anti-kinin TOP activity.

## Methods

### Mice and cell lineages

Six- to eight-week-old female C57BL/6 mice were obtained from the Center for Development of Experimental Models (CEDEME) animal facility, Federal University of São Paulo (UNIFESP), and kept in isolators, with autoclaved water and food. The animal experiments were carried out in accordance with the UNIFESP Ethics Committee for Animal Experimentation.

The B16F10 murine melanoma cell line is syngeneic in C57Bl/6 mice and was originally obtained from the Ludwig Institute for Cancer Research (São Paulo Branch). At the Experimental Oncology Unit (UNONEX), we isolated sublines from the original cell line with different phenotypes. The melanotic subline Nex2 (B16F10-Nex2) is characterized by low immunogenicity and moderate virulence. It forms lethal subcutaneous tumours, with no metastasis to the lung unless injected intravenously. The melanoma cells and human umbilical vein endothelial cells (HUVEC) were maintained in complete medium consisting of RPMI 1640, pH 7.2, supplemented with 10 mM N-2-hydroxyethylpiperazine-N'-2-ethanesulphonic acid (HEPES), 24 mM sodium bicarbonate, 10% heat-inactivated fetal calf serum (FCS) from Gibco (Minneapolis, MN, USA) and 40 μg/mL gentamicin sulfate (Hipolabor Farmacêutica, Sabará, MG, Brazil).

### Tumor cells growth and processing

B16F10-Nex2 cells were grown in 75-cm^3 ^flasks (Costar Corning, NY, USA) until 80–90% confluence. Spent 10% FCS-containing RPMI medium from melanoma cultures was replaced by serum-free RPMI medium (10 mL/flask) after three washings with PBS (10 mL/wash), following further incubation for 8 h. The culture supernatant fluid was collected, centrifuged at 1,800 *g *for 5 min and concentrated 10-fold using an Amicon cell (Millipore, MA, USA) with 12 kDa cut-off membrane and stirring with N_2 _positive pressure at 4°C. The concentrated culture medium (referred to as the 'supernatant') was used for measurements of enzymatic activity.

To prepare cell lysates and membranes, the cells were incubated for 8 h in serum-free medium and re-suspended in 50 mM Tris-HCl, pH 7.4. They were then lysed by sonication at 40 Hz (4 cycles of 60 s). After removal of the cell debris by centrifugation at 12,000 *g *for 5 min, the supernatant was centrifuged at 100,000 *g *for 2 h. The supernatant of this centrifugation represents the cell lysate and the pellet, re-suspended in 50 mM Tris-HCl, pH 7.4, represents the cell membrane preparation.

The protein content of samples was determined as previously described [34] using bovine serum albumin as standard. The culture concentrated supernatants, cell lysates and membrane preparations were used in proteolytic assays, in the in vitro angiogenesis assay on Matrigel and in Western blotting with anti-TOP and anti-neurolysin antibodies.

B16F10-Nex2 cells were irradiated at 10,000 rad and the Trypan Blue negative cells were used in the angiogenesis assay.

### Western blotting

For Western blotting of TOP and neurolysin, 10 μg of B16F10-Nex2 fractions (supernatant, lysate and membrane) were separated in 10% SDS-PAGE and then electrophoretically transferred onto nitrocellulose membrane (0.2 μm, Amersham Bioscience, England). Membranes were incubated for 1 h in PBS and 5% dry skim milk. The anti-TOP and anti-neurolysin antibodies (Proteimax, São Paulo, Brazil) at 1:1000 were used as the primary antibody, and the secondary antibody was horse-radish peroxidase-conjugated goat anti-rabbit antibody in PBS and 1% dry skim milk. The blot was visualized using the ECL detection system (Amersham Pharmacia Biotech).

### In vitro angiogenesis assay on Matrigel

BD Matrigel™ Matrix (B&D Biosciences, Bedford, MA, USA) was thawed on ice and then 15 μL per well was distributed in 96-well plates, and allowed to polymerize for 1 h at 37°C. HUVEC cells (5 × 10^3 ^cells/well) suspended in 100 μL of RPMI medium supplemented with 0.2% of FCS were added to each well in the presence of the following inducers or inhibitors, isolated or combined: BK (1 μM), B16F10-Nex2 supernatant, membrane preparation or lysate, rTOP (specific activity: 231 μM/min/mg protein), JA-2 (5 μM), NT (1 μM), Angiotensin-II (1 μM) or CA-074 (100 nM). In the co-culture model consisting of HUVEC cells and irradiated B16F10-Nex2 cells (5 × 10^3 ^cells/well), the tumor and endothelial cells were added together to Matrigel after polymerization. The co-culture assay was standardized with live irradiated melanoma cells to prevent tumor growth.

The plates were incubated at 37°C for 18 h and then images were captured at 8× magnification with a Sony Cyber-shot camera coupled to a light inverted microscope. The number of angiogenic structures (closed rings) was counted from 4 different wells, and the average value was determined for each sample. As a control of the assay HUVEC cells were plated on Matrigel without any addition.

### Peptides

FRET peptides derived from neurotensin (NT) and bradykinin (BK) were synthesized by the solid phase and classical solution methods of peptide synthesis [35,36] using *o*-aminobenzoic acid (Abz) as fluorescent group and ethylenediamino-2,4-dinitrophenyl (EDDnp) as fluorescence quencher, attached respectively to the N- and C-terminal groups of the peptides. All the obtained peptides were purified by semi-preparative HPLC on an Econosil C-18 column. The molecular mass and purity of synthesized peptides (94% or higher) were checked by MALDI-TOF mass spectrometry, using a TofSpec-E from Micromass, Manchester, UK. Nonderivatized NT and BK peptides were purchased from Sigma, St Louis, MO, USA.

### Kinetic assays

Hydrolysis of the fluorogenic peptidyl substrates (approx. 20 μM) at 37°C in 50 mM Tris-HCl buffer, pH 7.4, was followed by measuring the fluorescence at λ_em. _= 420 nm and λ_ex. _= 320 nm in a Hitachi F-2000 spectrofluorometer. The 1-cm-path-length cuvette containing 1 ml of the substrate solution was placed in a thermostatically controlled cell compartment for 5 min before the samples were added and the increase in fluorescence with time was continuously recorded for 5–10 min. The readings were converted into moles of hydrolyzed substrate per minute based on the fluorescence curves of standard peptide solutions before and after total enzymatic hydrolysis. The concentration of the peptide solutions was obtained by colorimetric determination of the 2, 4-dinitrophenyl group (17,300 M^-1^.cm^-1 ^extinction coefficient at 365 nm). The sample concentration for initial rate determination was chosen at a hydrolysis level less than 5% the substrate present. Inhibitors were added to the reactions to determine the putative contribution of various proteases in the cleavage of substrates using as control the inhibition by the same inhibitors of the recombinant enzymes.

PMSF, E-64, *o*-phenanthrolin, Z-Pro-Prolinal, leupeptin, captopril and S-nitroso-N-acetylpenicillamine (SNAP) were purchased from Sigma (St Louis, MO, USA). Bestatin was a gift from Kaethy B. Alves, UNIFESP, Brazil. The JA-2 inhibitor [37], originally from Ian Smith of the Baker Heart Research Institute, Australia, was provided by A.C.M. Camargo, Butantan Institute, Brazil. The specific antibodies against TOP and neurolysin were purchased from Proteimax (São Paulo, SP, Brazil). The results were recorded as the percentage of residual activity relative to control reactions run simultaneously in the absence of the inhibitor.

### Hydrolysis of neurotensin and bradykinin

The reactions of NT (pELYENKPRRPYIL) or BK (RPPGFSPFR) (20 μM each) with mammalian recombinant enzymes and B16F10-Nex2 supernatant, with or without inhibitors, were carried out for 1 h at 37°C in 50 mM Tris-HCl buffer, pH 7.4. Each aliquot of reaction products was then analyzed by HPLC, monitoring the absorbance at 220 nm.

### Determination of cleaved peptide bonds

The sites of peptide cleavage were identified by isolation of the fragments in analytical HPLC. Fractions were monitored by UV absorbance at 220 nm and fluorescence readings at λ_em. _= 420 nm and λ_ex. _= 320 nm. The retention times of the fragments produced were compared with authentic synthetic peptide sequences and molecular mass determination by MALDI-TOF (TofSpec-E, Micromass) mass spectrometry.

### Cloning and expression of recombinant enzymes

TOP and neurolysin genes were cloned from B16F10-Nex2 melanoma cells and expressed as indicated below. The expression vectors pHis3-TOP and pHis3-Neurolysin were constructed by insertion of the genes into pHis3 plasmid, a modified pET vector. The cDNAs encoding the full length murine melanoma TOP and neurolysin were reverse-transcribed from total B16F10-Nex2 melanoma RNA with superscript II-reverse transcriptase (Gibco BRL). The PCR mixture consisted of 1/10 of reaction-cDNA, 200 μM deoxynucleoside triphosphates, 2 mM MgCl_2_, 50 mM KCl, 20 mM Tris-HCl (pH 8,4), 1 U *taq *DNA polymerase and 50 pmoles of each of the primers: 5'-ATGAAGCCCCCCGCAG-3' and 5'-TCAGCACGCAGGCGCCTC-3' for TOP, and 5'-ATGATCACCCTGTGCC-3' and 5'-TTACGAAGCATTCAGGCC-3' for neurolysin. The PCR temperature cycle was 94°C for 3 min, followed by 35 cycles of 94°C for 1 min, 60°C for 1 min, 72°C for 3 min, and finally holding for 10 min at 72°C. The amplified fragments were recovered from 1% Agarose gel using BIOCLEAN for purification of DNA bands (BIOTOOLS, Brazil) and cloned on pGEM-T easy vector (PROMEGA, Madison, USA). The cloned gene fragment was excised from the plasmid by digestion with *Eco*RI (Invitrogen, Carlsbad, CA, USA) and ligated into the *Eco*RI site of pHis3 vector. The resulting constructs were verified by restriction enzyme mapping and DNA sequencing. The gene sequences were translated and compared with mouse TOP and neurolysin published sequences.

For expression, *E. coli *BL21 (DE3) pLysS was transformed by heat shock in expression vectors and these were grown at 37°C for 16 h, with shaking, in Luria-Bertani medium, with ampicillin (100 μg/ml) and chloramphenicol (50 μg/ml). The transformed bacteria were re-inoculated in fresh medium and grown with antibiotic selection to A_600 _= 0.6, and the expression of the recombinant protein was induced with 1 mM isopropyl β-D-thiogalactoside (IPTG), for 4 h. Bacterial cultures were centrifuged at 1,075 *g *for 20 min at 4°C, re-suspended in 50 mM Tris-HCl, pH 7.4 and then lysed by sonication at 40 Hz (4 cycles of 60 s). After removal of the bacterial debris by centrifugation, the supernatants were incubated with Ni-NTA (Nickel-nitrilotriacetic acid) Agarose (Qiagen, Hilden, Germany) equilibrated in Buffer A (20 mM Tris-HCl, pH 8.5, 100 mM KCl, 20 mM imidazole, 10 mM 2-mercaptoethanol, 10% [v/v] glycerol) for 3 h at 4°C with shaking. After washing with 10 volumes of Buffer A, recombinant protein was eluted from the column with 2 volumes of Buffer C (20 mM Tris-HCl, pH 8.5, 100 mM KCl, 100 mM imidazole, 10 mM 2-mercaptoethanol, and 10% [v/v] glycerol). Recombinant protein was desalted in a PD-10 column (Amersham Pharmacia Biotech), analyzed by polyacrylamide gel electrophoresis after staining with Coomassie blue R-250 (Bio-Rad Laboratories, Richmond, CA, U.S.A.) and used in the assays below.

### Cell proliferation assay

B16F10-Nex2, 5 × 10^3 ^cells per well, was cultivated in 96-well plates and, after cell attachment for 6 h, incubated for 12, 24 and 48 h in the presence or absence of JA-2 (3 μM) and/or bestatin (50 μM) inhibitors. The cell proliferation was measured using the Cell Proliferation Kit I (MTT) (Boehringer Mannheim), an MTT-based colorimetric assay for quantification of cell proliferation and viability. Readings were made in a plate reader at 570 nm.

### In vivo grafting of tumor cells

For in vivo experiments, C57BL/6 mice (4–5 animals per group) were inoculated subcutaneously on the right flank with a suspension containing 5 × 10^4 ^viable cells in 0.1 mL of serum-free RPMI medium in the presence or absence of active or inactive rTOP (8 μg protein and specific activity of 231 μM/min/mg protein). Tumor growth was followed by measuring its volume with a caliper three times a week, and also scoring the survival of challenged animals. The tumor volume was calculated using the formula: *V *= 0.52 × D_1_^2 ^× D_3_, where D_1 _and D_3 _are the short and long tumor diameters, respectively. Maximal volumes of 3–4 cm^3 ^were allowed before sacrifice. Survivals of mice were scored and statistically compared.

### Statistical analysis

The data are represented as means ± SE. Statistical significance was determined by the Student's *t *test. All experiments were conducted two or more times. Reproducible results were obtained and representative data are shown. The survival plots of animals challenged with tumor cells and injected simultaneously with active or inactive rTOP were analyzed by Kaplan-Meier log rank test.

## Results

### Effect of B16F10-Nex2 melanoma cells on angiogenesis

**Figure 1:**
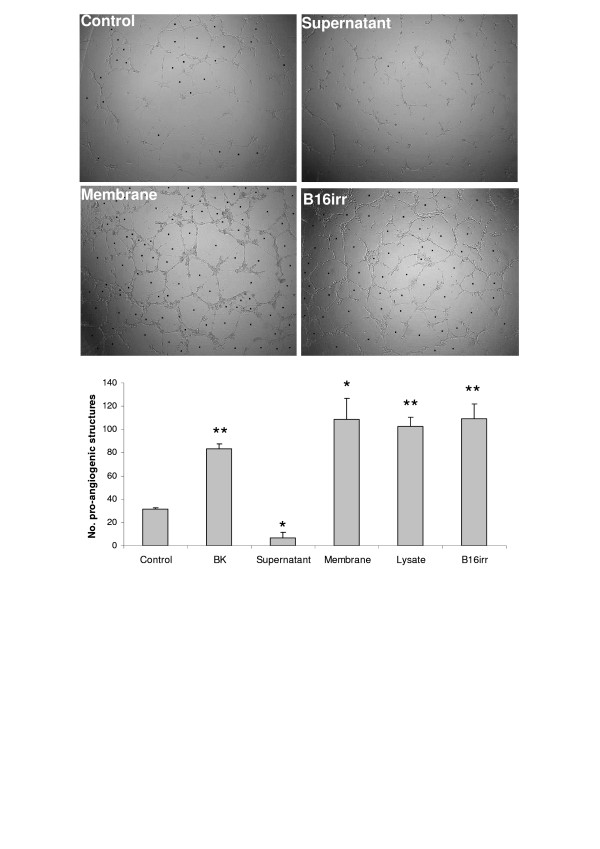
**Effect of BK and B16F10-Nex2 tumor cells on pro-angiogenic closed structures formed by sprouting of endothelial cells**. HUVECs were plated on Matrigel in medium supplemented with 0.2% of FCS in the presence of BK, B16F10-Nex2 supernatant, cell membrane or lysate and irradiated B16F10-Nex2 whole cells. The number of pro-angiogenic structures was counted after 18 h. One representative picture of four different treatments is shown with the respective counts. * *p *< 0.005 vs control; ** *p *< 0.0005 vs control.

The effect of B16F10-Nex2 melanoma cells on endothelial cell angiogenesis was examined in sets of co-culture on Matrigel, as an angiogenesis assay. HUVEC endothelial cells were plated on Matrigel with BK, irradiated B16F10-Nex2 cells or B16F10-Nex2 supernatant, lysate or membrane preparation (Fig. 1). The growth of HUVECs on Matrigel with 0.2% fetal calf serum led to the formation of closed intercellular compartments arising from endothelial cell sprouting (pro-angiogenic structure) independent of any other factor. Addition of BK to the incubation mixture at 1 μM stimulated formation of these structures more than 2-fold. The co-culture of irradiated melanoma and endothelial cells increased the number of pro-angiogenic structures to the same level as in BK-treated HUVECs. The same occurred with the B16F10-Nex2 membrane preparation and lysate. In contrast, B16F10-Nex2 supernatant exerts a clear negative effect on angiogenesis compared to the control.

**Figure 2:**
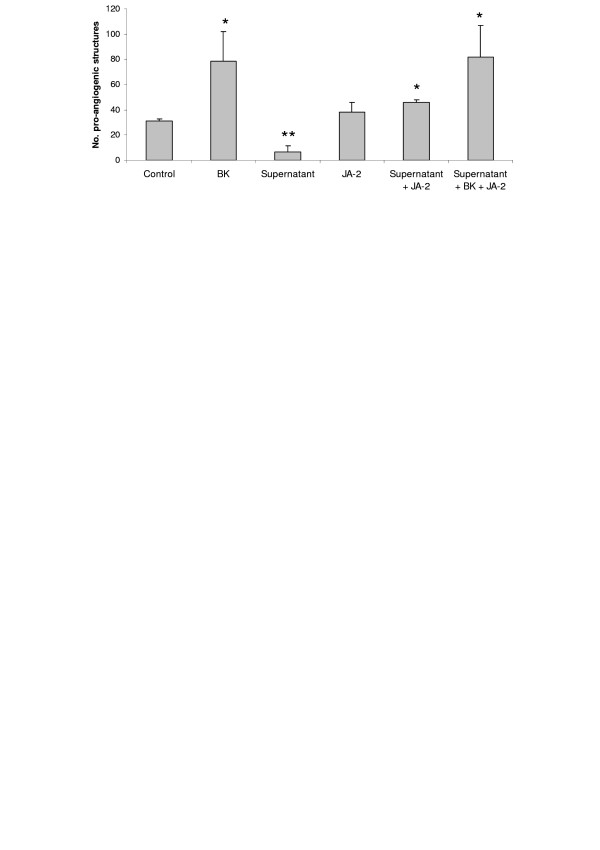
**Effect of JA-2 on in vitro Matrigel angiogenesis assay**. HUVECs were plated on Matrigel in medium supplemented with 0.2% of FCS in the presence of BK, B16F10-Nex2 supernatant and JA-2 (thimet oligopeptidase inhibitor). The number of pro-angiogenic structures was counted after 18 h. * *p *< 0.05 vs control; ** *p *< 0.005 vs control.

To characterize the angiogenesis inhibitory factors secreted in the supernatant of B16F10-Nex2 cells, we tested the effect of JA-2, an inhibitor of thimet oligopeptidase. This enzyme is able to hydrolyze BK, a known pro-angiogenic factor. We observed that JA-2 reversed the negative effect promoted by the melanoma supernatant, but did not affect the basal formation of pro-angiogenic structures (Fig. 2).

### Enzymatic activity of B16F10-Nex2 supernatant

To further address the enzymatic activity of B16F10-Nex2 supernatant, FRET peptides derived from NT and BK, previously employed to determine the specificity of recombinant TOP and neurolysin oligopeptidases [38,39], were used. The melanoma supernatant was able to cleave all the assayed substrates, except Abz-GFPPFRQ-EDDnp and Abz-rRL-EDDnp (Table 1), which were also resistant to recombinant TOP and neurolysin. The fluorogenic substrate Abz-rRL-EDDnp was previously used to identify neprilisin oligopeptidase [40], which was not detected in the B16F10-Nex2 supernatant. However, the conditioned medium cleaved preferentially Abz-GFSPFRQ-EDDnp, which is a very susceptible substrate for TOP.

**Table 1:** Proteolytic activity of B16F10-Nex2 supernatant and recombinant enzymes on FRET peptides

**Abz-peptidyl-EDDnp**	**Specific activity (nmoles/min/mg protein)**		
	
	**B16F10-Nex2**	**rTOP**	**rNeurolysin**
GFSPFRQ	2.7 ± 0.6	100 ± 9.8	5.5 ± 1.0
GFSPFR	0.6 ± 0.1	21 ± 4.2	4.4 ± 0.5
GFSIFRQ	0.6 ± 0.1	14 ± 4.4	1.6 ± 0.2
GFPPFRQ	0	0	0
NKPRRPQ	0.4 ± 0.4	29 ± 3.3	58 ± 8.3
RPPGFSPFRQ	1.8 ± 0.1	51 ± 5.5	8.8 ± 0.7
rRL	0	0	0

B16F10-Nex2 supernatant and recombinant enzymes were incubated with fluorogenic substrates (20 μM) in 50 mM Tris-HCl, pH 7.4 at 37°C. Hydrolysis was followed by measuring the fluorescence at λem. = 420 nm and λex. = 320 nm. Results are expressed as means ± SD.

**Figure 3:**
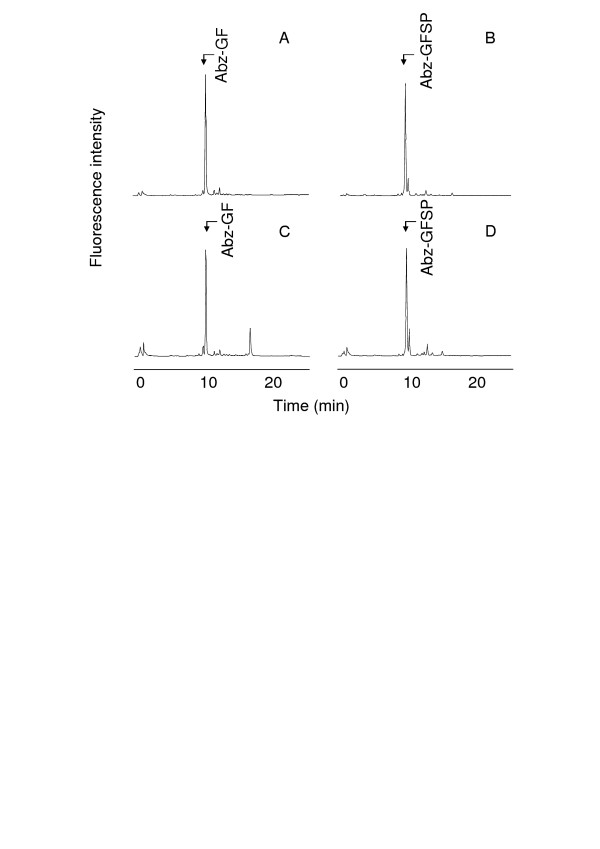
**HPLC analysis of FRET peptides degradation by the B16F10-Nex2 supernatant**. Abz-GFSPFR-EDDnp (A, C) or Abz-GFSPFRQ-EDDnp (B, D) were incubated with recombinant oligopeptidase TOP (A, B), or B16F10-Nex2 supernatant (C, D) in 50 mM Tris-HCl pH 7.4 at 37°C. Reaction products were separated by HPLC and were identified by mass spectrometry. Chromatograms developed by fluorescence detection at λ_em. _= 420 nm and λ_ex. _= 320 nm.

HPLC analyses of Abz-GFSPFR-EDDnp and Abz-GFSPFRQ-EDDnp degradation products by B16F10-Nex2 supernatant and rTOP are shown in Figure 3. As previously described for TOP and neurolysin [39], Abz-GFSPFR-EDDnp was cleaved at Phe-Ser bond, but the presence of glutamine in Abz-GFSPFRQ-EDDnp shifted the cleavage to Pro-Phe bond (Fig. 3A and 3B). Melanoma supernatant hydrolyzed these substrates as the recombinant TOP and neurolysin do (Fig. 3C and 3D), indicating that the melanoma peptidase activities could be related to these enzymes.

### Inhibition of the peptidase activity

The effects of the peptidase inhibitors on the hydrolysis of Abz-GFSPFRQ-EDDnp by B16F10-Nex2 supernatant, recombinant TOP and neurolysin are shown in Table 2. PMSF (0.1 mM), E-64 (0.1 mM), Z-Pro-Prolinal (1 μM), leupeptin (0.2 mM) and captopril (20 μM) have no effects. Pro-Ile (1 mM), a specific inhibitor of neurolysin [41], inhibited by ~40% the B16F10-Nex2 supernatant enzymes and ~50% the recombinant neurolysin, but does not inhibit TOP. *o*-Phenanthrolin (4 mM) and JA-2 (3 μM) were the most effective inhibitors of both, the standard recombinant enzymes and of the B16F10-Nex2 supernatant. *o*-Phenanthrolin is a specific inhibitor of metallo-proteases and JA-2 was described as a specific inhibitor of TOP [37], but it also inhibits neurolysin as we have shown here. The activities of B16F10-Nex2 supernatant on fluorogenic quenched substrates are shown in Table 1, which were also totally inhibited by *o*-phenanthrolin and JA-2.

**Table 2:** Effect of inhibitors on the hydrolysis of Abz-GFSPFRQ-EDDnp by B16F10-Nex2 supernatant and recombinant TOP and Neurolysin

**Inhibitors**	**Relative hydrolysis (%)^1^**		
	
	**B16F10-Nex2**	**rTOP**	**rNeurolysin**
Control (no inhibitor)	100	100	100
*o*-phenanthrolin (4 mM)	0	0	0
JA-2 (3 μM)	2.6 ± 2.1	0.6 ± 1.2	1 ± 1
Pro-Ile (1 mM)	60 ± 6	87 ± 5	52 ± 4
PMSF (0.1 mM)	94 ± 6	91 ± 4	93 ± 4
E64 (0.1 mM)	88 ± 3	98 ± 2	99 ± 2
Leupeptin (0.2 mM)	81 ± 6	96 ± 2	96 ± 4
Z-Pro-Prolinal (1 μM)	93 ± 1	88 ± 3	92 ± 4
Captopril (20 μM)	92 ± 3	97 ± 2	98 ± 2

^1^100% hydrolysis represents the cleavage of 20 μM of Abz-GFSPFRQ-EDDnp by B16F10-Nex2 supernatant and recombinant enzymes during 5 min at 37°C in 50 mM Tris-HCl, pH 7.4. The values are means of three independent experiments.

### Hydrolysis of neurotensin and bradykinin

**Figure 4:**
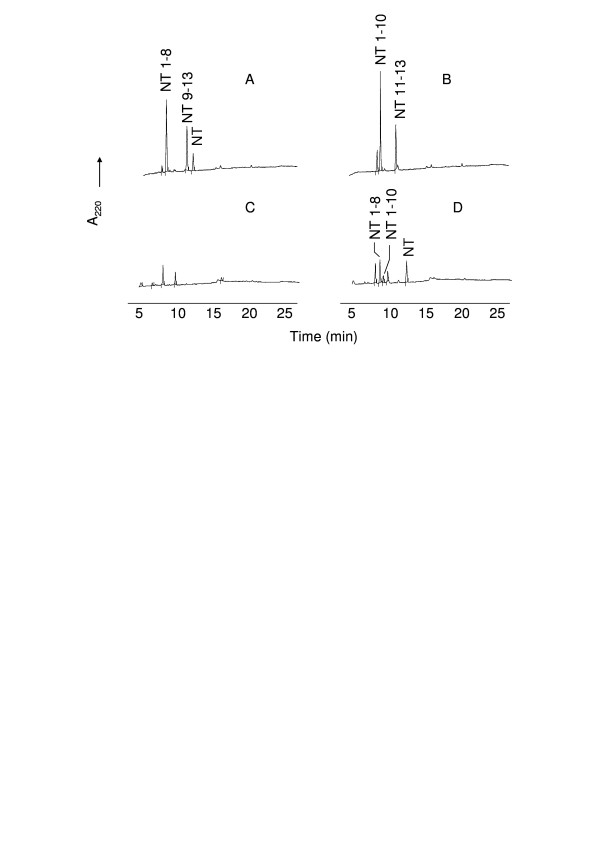
**HPLC analysis of neurotensin (NT) degradation by B16F10-Nex2 supernatant**. Neurotensin (20 μM) was incubated for 1 h at 37°C with recombinant TOP (A), neurolysin (B) or B16F10-Nex2 supernatant (D) in 50 mM Tris-HCl, pH 7.4. The reaction products were separated by HPLC. HPLC profile of melanoma supernatant without NT is shown on panel C. The neurotensin fragments were determined by mass spectrometry.

**Figure 5:**
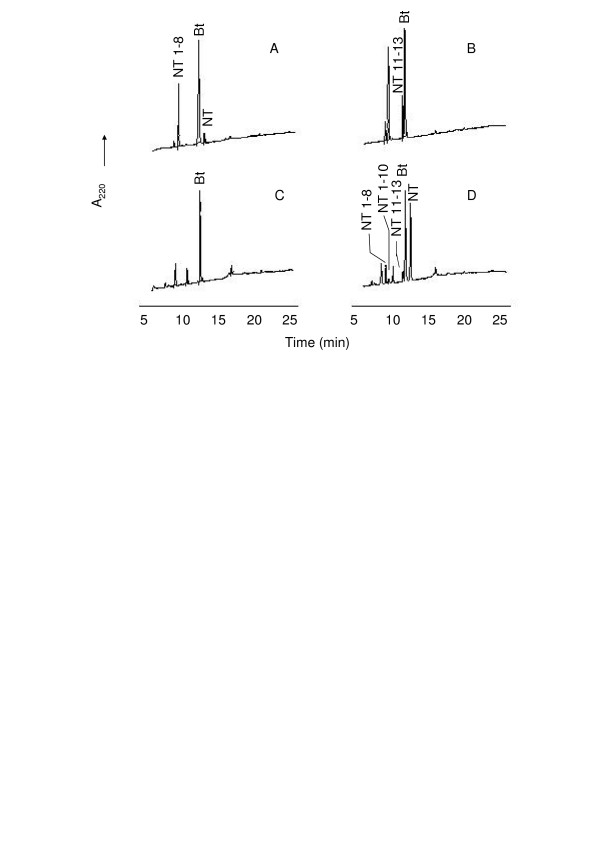
**HPLC analysis of neurotensin (NT) degradation by B16F10-Nex2 supernatant in the presence of bestatin**. Neurotensin (20 μM) was incubated for 1 h at 37°C with recombinant TOP (A), neurolysin (B) or B16F10-Nex2 supernatant (D) in the presence of bestatin in 50 mM Tris-HCl, pH 7.4. The reaction products were separated by HPLC. HPLC profile of melanoma supernatant in the presence of bestatin without NT is shown on panel C. The neurotensin fragments were determined by mass spectrometry.

NT represents the only known peptide differentially cleaved by TOP and neurolysin, therefore it is a very useful reagent to distinguish these peptidase activities. TOP hydrolyzes the Arg-Arg bond, producing NT_1–8 _and NT_9–13 _whereas neurolysin cleaves the Pro-Tyr bond, producing NT_1–10 _and NT_11–13_. Incubation of NT with melanoma supernatant resulted in the generation of NT_1–8 _and NT_1–10 _(Fig. 4D), the same N-terminal fragments generated respectively by cleavage of NT by TOP and neurolysin (Fig. 4A and 4B), that were identified by mass spectrometry. The same assay was performed in the presence of bestatin (50 μM), an aminopeptidase inhibitor. In previous assays, bestatin was able to completely inhibit the supernatant peptidase activity using Phe-MCA as substrate, indicating the presence of an aminopeptidase in the melanoma supernatant (data not shown). In the presence of bestatin, the fragment of NT_10–13 _(Fig. 5C) was detected as confirmed by mass spectrometry. The aminopeptidase presents in the B16F10-Nex2 supernatant was responsible for cleaving the NT C-terminal fragments. The fragment NT_9–13 _was not observed in the HPLC due to the presence of bestatin at exactly the same elution time, as shown in Figure 5A. Bestatin, as expected, was unable to modify the HPLC profile of TOP and neurolysin (Fig. 5A and 5B).

**Figure 6:**
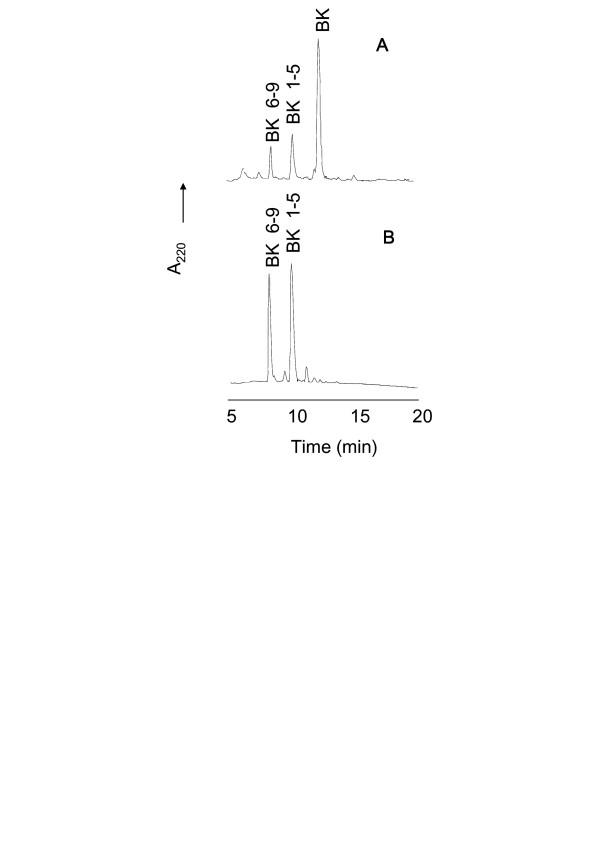
**HPLC analysis of BK degradation by B16F10-Nex2 supernatant**. BK (20 μM) was incubated for 1 h at 37°C with recombinant TOP (A) or B16F10-Nex2 supernatant (B) in 50 mM Tris-HCl, pH 7.4. The reaction products were separated by HPLC. Bradykinin fragments were determined by mass spectrometry.

The cleavage of BK at the Phe-Ser bond by TOP and neurolysin was previously described [42]. The B16F10-Nex2 supernatant cleaved BK at the same site, with the corresponding fragments being identified by mass spectrometry (Fig. 6B). The HPLC of fragments arisen from BK hydrolysis by recombinant TOP was the control shown in Figure 6A.

### Cloning of TOP and neurolysin from B16F10-Nex2 melanoma cells

Both melanoma enzyme cDNAs were cloned, confirming the presence of TOP and neurolysin in melanoma cells, and the gene and translated protein sequences were compared to the mouse sequences (data not shown).

The cDNA sequence of TOP from melanoma showed a single nucleotide change by comparing with the mouse enzyme. The point mutation on adenine 300 to guanine was not able to cause alterations in the amino acid sequence.

The complete sequencing of neurolysin cDNA from melanoma showed three modifications in relation to the mouse gene: thymine 666 to cytosine, thymine 1268 to cytosine and thymine 1316 to cytosine. Only two changes promoted substitutions in the amino acid sequence: Val 423 to Ala and Leu 439 to Pro. These changes did not seem to affect the active site of the enzyme and did not include amino acids described as important for enzyme activity [43,44].

### Peptidase activity in the culture supernatant, lysate and membrane preparation of B16F10-Nex2 cell line

The oligopeptidase activities in the culture supernatant, lysate and membrane preparations of B16F10-Nex2 melanoma cells were compared. The melanoma cells display the greatest oligopeptidase activity in the lysate as shown by hydrolysis of Abz-GFSPFRQ-EDDnp in Table 3. The activity in the supernatant is 10-fold less that of the lysate, when the results are expressed in enzyme activity per cell. If the assay is standardized by specific activity, the supernatant and lysate values were 1.39 and 3.22 μM/min per mg of protein, respectively, because the protein content in the lysate is approximately 4-fold that in the supernatant. The total activity based on the volume of the fractions showed that the lysate had much more oligopeptidase activity than the supernatant. The activity in the membrane fraction was very low, but the protein was clearly detected by Western blotting (data not shown). To demonstrate the expression levels of TOP and neurolysin in melanoma cells, B16F10-Nex2 fractions were submitted to immunoblotting, using specific antibodies against these two oligopeptidases. We detected TOP expression in the supernatant, lysate and membrane preparations of B16F10-Nex2 melanoma cells (data not shown). Neurolysin expression was not detected using this method. The specific anti-TOP antibody inhibited 80% of the B16F10-Nex2 catalytic activity, using the FRET peptide Abz-GFSPFRQ-EDDnp, whereas the anti-neurolysin antibody inhibited only 20% of the melanoma enzyme activity (Table 3).

**Table 3:** Oligopeptidase activities in the culture supernatant, cell lysate and membrane fraction of B16F10-Nex2 cells

**Cell fraction**	**Specific activity (μM/min per mg)**	**Activity per cell (pM/min per 10^3 ^cells)**	**Total activity (μM/min)**
Supernatant	1.39 ± 0.24	36 ± 16	3.36 ± 0.15
Lysate	3.22 ± 0.96	326 ± 15	31.56 ± 0.16
Lysate + Anti-TOP Ab	0.64 ± 0.19	65 ± 5	6.31 ± 0.03
Lysate + Anti-Neurolysin Ab	2.58 ± 0.77	261 ± 5	25.25 ± 0.13
Membrane*	< 0.01	3 ± 0.5	0.2 ± 0.01

Culture supernatant, cell lysate and membrane preparation of B16F10-Nex2 cells were incubated with the fluorogenic substrate Abz-GFSPFRQ-EDDnp (20 μM) in 50 mM Tris-HCl, pH 7.4 at 37°C. Enzyme activity was followed by measuring the fluorescence at λem. = 420 nm and λex. = 320 nm. Results are expressed as means ± SD. Ab= antibodies, used at 1:300.

*Although the activity of TOP in the membrane was low, the protein was clearly detected by Western blotting with anti-TOP antibody.

### Tumor cell proliferation assay

**Figure 7:**
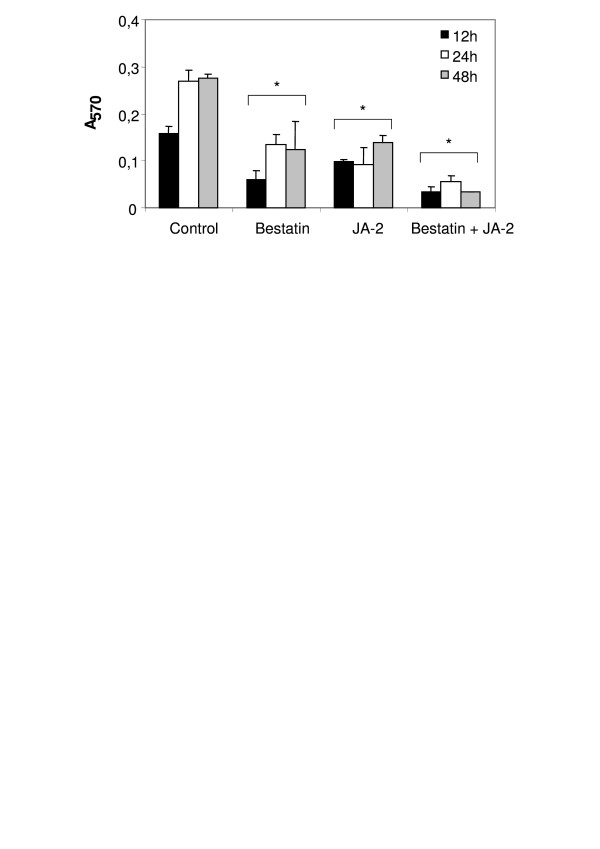
**B16F10-Nex2 proliferation assay in the presence of JA-2 and/or bestatin inhibitors**. 5 × 10^3 ^B16F10-Nex2 cells were cultivated in 96-well plates, incubated for 12, 24 and 48 h with JA-2 (3 μM) and/or bestatin (50 μM) inhibitors, and the cell proliferation was measured using MTT in comparison with Controls. *p < 0.05.

A proliferation assay in vitro was carried out in the presence of JA-2 and bestatin and the results are shown in Figure 7. JA-2 and bestatin decreased B16F10-Nex2 cell growth 50% and 56% respectively in 48 h. A synergistic inhibitory effect (88% inhibition of proliferation in 48 h) was observed when both inhibitors were added together in the melanoma culture.

### Effect of TOP on tumor development in vivo

To assess the effect of TOP on the tumor development in vivo, tumor cells were injected subcutaneously in syngeneic mice together with active or inactive rTOP at concentrations that did not affect the growth of tumor cells in vitro.

**Figure 8:**
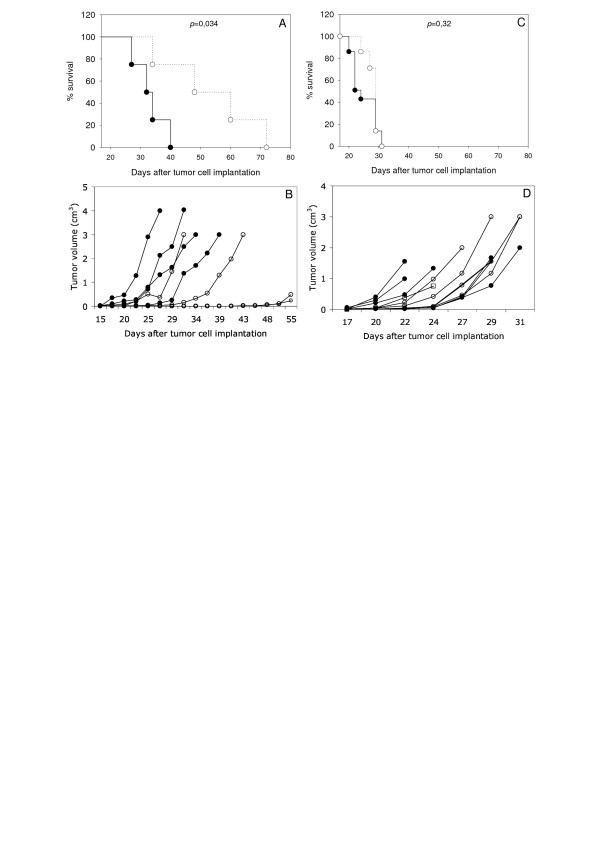
**Effect of active and inactive rTOP on tumor cell development and animal survival after subcutaneous implantation of B16F10-Nex2 melanoma cells**. 5 × 10^4 ^viable cells were injected subcutaneously with 8 μg of active rTOP (A, B, open circle), inactive rTOP (C, D, open circle), and PBS (control, solid circle) in C57Bl/6 mice (4–5 animals per group). The tumor volume was measured every 2–3 days and a maximal volume of 3 cm^3 ^was allowed before sacrifice. (B, D), tumor volume of individual animals; (A, C), survival plots. Statistical analysis of survivals was performed using Kaplan-Meier test.

Tumor cell implantation in the presence of 8 μg of active rTOP (specific activity: 231 μM/min per mg) was followed by delayed tumor growth and prolonged survival of injected mice (*p *= 0.034) (Fig. 8A and 8B). In contrast there was no significant difference (*p *= 0.32) in the tumor development when B16F10-Nex2 cells were injected in the presence of inactive rTOP (Fig. 8C and 8D).

### Effect of TOP in angiogenesis assay on Matrigel

**Figure 9:**
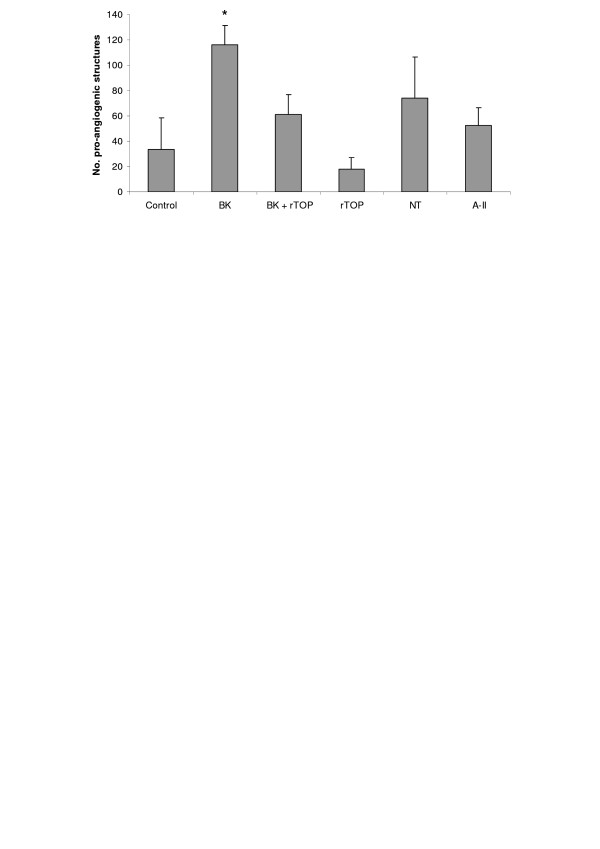
**Effect of rTOP on in vitro Matrigel angiogenesis assay**. HUVECs were plated on Matrigel in medium supplemented with 0.2% of FCS in the presence of BK (1 μM), rTOP (specific activity: 231 μM/min/mg), BK+ rTOP, NT (1 μM) and A-II (1 μM). The number of pro-angiogenic structures was counted after 18 h. * *p *< 0.05 vs control.

We examined the ability of rTOP to stimulate pro-angiogenic structures by HUVECs cultured on Matrigel. These structures arise from sprouting of endothelial cells and formation of closed intercellular compartments that can be quantified. As shown in Figure 1, BK stimulated the formation of pro-angiogenic structures >2-fold. TOP alone was able to inhibit endothelial cell extensions and reverse the angiogenic stimulus promoted by BK (Fig. 9). Further, NT and angiotensin II (A-II), other peptides hydrolyzed by TOP, also stimulated angiogenesis.

### Inhibition of rTOP activity by SNAP

To verify the possible inhibition of rTOP by nitric oxide, which could suggest a way of controlling the activity of the endo-oligopeptidase, we carried out a kinetic assay with the recombinant enzyme and S-nitroso-N-acetylpenicillamine (SNAP), as NO donor. SNAP (1 mM to 100 μM) was incubated with rTOP for 10 minutes and then the fluorogenic substrate Abz-GFSPFRQ-EDDnp was added to the reaction. The result in Table 4 shows that SNAP inhibited TOP hydrolytic activity using substrate Abz-GFSPFRQ-EDDnp, in a dose-dependent manner, but not time-dependent kinetics (data not shown). At 100 μM, SNAP was unable to inhibit TOP. Addition of 1, 4-dithiothreitol (DTT) in the assay before the fluorogenic substrate prevented TOP inhibition by SNAP.

**Table 4:** Effect of the NO donor SNAP on the hydrolysis of Abz-GFSPFRQ-EDDnp by rTOP

**SNAP**	**Hydrolysis (%)**^1^
Control (no inhibitor)	100
1 mM	38 ± 3
500 μM	50 ± 3
250 μM	83 ± 4
100 μM	98 ± 2
1 mM + DTT 0.5 mM	100 ± 1

^1^100% hydrolysis represents the cleavage of 20 μM of Abz-GFSPFRQ-EDDnp by rTOP during 5 min at 37°C in 50 mM Tris-HCl, pH 7.4.

## Discussion

We describe in the present work the stimulating effect on angiogenesis of irradiated melanoma B16F10-Nex2 tumor cells using HUVEC on Matrigel substrate. A melanoma cell lysate and membrane preparation showed the same effect of whole irradiated tumor cells, suggesting a distribution of pro-angiogenic factors in both membrane and cytoplasm of the tumor cells. In contrast, B16F10-Nex2 conditioned medium inhibited endothelial cell sprouting and the formation of pro-angiogenic structures. The negative effect on angiogenesis was reversed by the thimet oligopeptidase inhibitor JA-2, suggesting the anti-angiogenic role of this or a similar secreted oligopeptidase into the melanoma culture supernatant.

The presence of TOP and neurolysin in melanoma cells and culture supernatants was confirmed by hydrolytic assays and by cloning and sequencing the corresponding cDNAs from B16F10-Nex2 cells. The hydrolysis of NT, BK and FRET peptides were consistent with the presence of Zn-dependent oligopeptidases with a catalytic activity similar to that of both TOP and neurolysin. In addition, the enzymes of the melanoma supernatant hydrolyzed the substrate Abz-GFSPFRQ-EDDnp at the Pro-Phe bond as described for TOP and neurolysin, indicating that the melanoma activity had the same specificity as of the recombinant mouse enzymes (Figs 3C and 3D).

The complete inhibition of melanoma peptidase by *o*-phenanthrolin as well as by JA-2, and the partial inhibition by Pro-Ile give support to the functional similarity of melanoma enzymes with the oligopeptidases TOP and neurolysin. The absence of inhibition of peptidase activity by PMSF, E-64, Z-Pro-Prolinal, leupeptin and captopril excludes the presence of other peptidase classes eventually responsible for the cleavage of Abz-GFSPFRQ-EDDnp, the substrate used in these assays. The generation of NT_9–13 _and NT_10–13 _in the presence of bestatin suggested that an aminopeptidase was the most likely enzyme responsible for degradation of the C-terminal fragment of NT.

The cDNA sequencing of melanoma enzymes showed few mutations in comparison with the mouse counterparts. The only modification found in the melanoma TOP gene sequence did not cause an alteration in the amino acid sequence. The melanoma neurolysin gene showed three modifications in relation to mouse gene sequence, but only two changes promoted substitutions in the amino acid sequence away from the conserved Zn-binding catalytic site (HEFGH) or from other amino acids described as important to enzyme activity [43,44].

We show here that significant TOP and neurolysin-like activities can be detected in conditioned media, lysate and membrane preparations from melanoma cells. The oligopeptidase activity per cell was 10-fold greater in the lysate than supernatant. The activity in the cell membrane preparation was scarce. When the assay was normalized by specific activity, the supernatant and lysate values were similar, since the protein content in the lysate is approximately 4-fold that in the supernatant. Using specific antibodies against the studied oligopeptidases, we showed that TOP activity in melanoma cells is significantly greater than that of neurolysin. TOP expression as detected by immunoblotting was prominent in the supernatant, lysate and membrane fraction of B16F10-Nex2 cells.

The importance of the oligopeptidases for melanoma cells in vitro was assessed by proliferation assay in the presence of JA-2 and bestatin. We observed a decreased B16F10-Nex2 cell proliferation in the presence of JA-2, bestatin and a greater inhibition with both. Presumably both inhibitors may act intracellularly, inhibiting oligopeptidases and aminopeptidases that are essential for melanoma cells. The secretion of both enzymes, TOP and neurolysin, might, however, influence tumor growth in vivo by affecting angiogenesis. Mice injected with active rTOP at a concentration that was not toxic in vitro, showed a delay in tumor development and increased survival of animals compared with mice challenged with tumor cells and inactive rTOP, indicating that the active enzyme and not only the protein was necessary for this effect. A possible target of rTOP activity in the tumor microenvironment could be kinins stimulating angiogenesis.

Tumors require an adequate supply of oxygen, metabolites and an effective way to remove waste products [45]. The generation of new blood vessels for tumor blood supply is thus a rate-limiting step in tumor progression, being a prerequisite for the rapid clonal expansion associated with the formation of macroscopic tumors [46]. Experimental data suggest that the angiogenic stimulation [46,47] is activated during the early stages of tumour development [48-51]. During development of cutaneous melanoma in humans a similar stage-specific stimulation is also evident [52]. Tumors appear to activate the angiogenic switch by changing the balance of angiogenesis inducers and counteracting inhibitors, so that tumor neovascularization and consequent growth depends on how heavily the balance tips towards angiogenesis [53,54]. One strategy for shifting the balance involves proteases that can control the bioavailability of angiogenic activators and inhibitors.

A prototype of pro-angiogenic molecule is bradykinin. Evidence suggests that BK may be one of the primary mediators responsible for tumor angiogenesis and, consequently, of tumor growth [28-33]. It is well known that endothelial cells can synthesize and secrete tissue kallikrein [55]. Schmaier et al [56] have also shown the expression of high molecular weight kininogen in human umbilical vein endothelial cells (HUVEC). The generation of the vasoactive peptide bradykinin from HUVEC-bound high molecular weight kininogen is also known [57]. Therefore, endothelial cells, particularly those used in the present study (HUVEC) do produce kininogen and kallikrein and generate BK. Such functional activities of endothelial cells are stimulated in Matrigel. The inhibitory effect of active rTOP inoculated with tumor cells, could well involve the hydrolysis of bradykinin, shifting the balance of angiogenic/anti-antiangiogenic factors at an early stage of tumor implantation. In agreement with this we have shown in an angiogenesis assay with HUVEC on Matrigel that rTOP was able to reverse the angiogenic stimulation promoted by BK. Similarly to BK, NT and A-II, two peptides that are hydrolyzed by TOP, were able to stimulate the endothelial cell sprouting. These peptides are further examples of positive modulators of the angiogenic process.

The results suggest that TOP affects angiogenesis in vitro and in vivo, at concentrations that did not inhibit B16F10-Nex2 tumor cells directly. The secretion of TOP by tumor cells would therefore favor an anti-angiogenic response in balance with the pro-angiogenic stimuli by other tumor proteases expressed for instance at the cell surface and released in the medium. We incubated HUVEC cells with CA-074, a cathepsin B inhibitor, in the Matrigel angiogenesis assay and showed stimulation of angiogenesis. The same inhibitor enhanced the pro-angiogenic effect induced by B16F10-Nex2 lysate, tumor cell membrane, and in the co-culture of B16F10-Nex2 with HUVEC cells (data not shown). References to the role of cathepsin B on tumor angiogenesis are contradictory. In human tumors, there is evidence of a positive correlation between the level of cathepsin B and angiogenesis [58], and the inhibition of cathepsin B expression was associated with angiogenesis suppression [59]. In contrast, the generation of endostatin by cathepsin B could block the angiogenesis in many tumor systems [60]. The role of cathepsins in melanoma requires additional studies in face of the multienzymatic complex of the tumor microenvironment. Clearly, in the in vitro Matrigel invasion assays, cathepsins B and L increase the angiogenesis-independent invasive capacity of tumor cells [61]. We described a more invasive B16F10 clone (Nex2B) as compared to a less invasive one (2D), though with a greater capacity of lung colonization than 2B, based on the extracellular rather than intracellular accumulation of cathepsins B, D, and L [1]. Nevertheless, an intracellular role for cathepsin B in matrix degradation has been identified [62], and also, three forms of extracellularly active cathepsin B and two forms of active cathepsin L have been described in the highly invasive melanoma cell line MV3 [63]. These isoforms add to the complexity of the system and demand a careful study to unravel their role on tumor growth.

An additional control of angiogenesis and tumor growth may exist based on the local release of NO. Kashiwagi et al. [64] using intravital microscopy demonstrated in a B16 murine melanoma model that eNOS from vascular endothelial cells is the predominant generator of NO. NO released by endothelial cells could be an inhibitory ligand of TOP activity. The inhibition of enzymes by nitric oxide has been demonstrated in the cysteine proteases [65-67], but not metallo-proteases. TOP and dipeptidylpeptidase IV (DPP IV) were not inhibited by the NO donors sodium nitroprusside(SNP) and 3-morpholinosydnoimine (SIN-1) in the 1–100 μM range [68]. Both compounds, however, were shown to release peroxynitrite, a reactive nitrogen species that primarily can nitrate tyrosine residues [69]. Site-directed mutation of Tyr residues at positions 612 and 613 in TOP and neurolysin, respectively, strongly reduced *k*_*cat*_/*K*_*M *_for both enzymes [70].

Nitrosothiols, which may act as NO reservoirs, could nitrosate cysteine residues [71], such as those that play an important role in TOP activity [72]. Presently, we exposed rTOP to SNAP a nitrosothiol, and the activity of the enzyme was inhibited in a concentration-dependent manner (250 μM to 1 mM range). Such inhibition was abolished in the presence of DTT. This result should be considered in the perspective of a tumor microenvironment with hydrophobic niches that may increase the efficiency of NO-based regulatory reactions [73], potentially influencing angiogenesis and tumor growth.

## Conclusion

The present work describes TOP and neurolysin oligopeptidases in B16F10-Nex2 melanoma cells and shows the importance of these enzymes for tumor proliferation in vitro and in vivo. Recombinant TOP protected mice against the subcutaneous challenge with murine melanoma cells. The results suggest that secreted thimet oligopeptidase in tumor cells and bradykinin are two antagonist factors that may regulate or trigger the angiogenic switch essential for melanoma growth. A regulatory role of NO or S-nitrosothiols on TOP activity is suggested. Together with endogenous inhibitors, endo-oligopeptidases secreted by tumor cells can also regulate angiogenesis and might also be studied, in adequate protocols, as potential anti-tumor agents.

## Abbreviations

TOP or EC 24.15, metallo-endopeptidase EC 3.4.24.15; EC 24.16, metallo-endopeptidase EC 3.4.24.16; rTOP, recombinant thimet oligopeptidase; FRET, fluorescence resonance energy transfer; HUVEC, human vein endothelial cell; FCS, fetal calf serum; NT, neurotensin; BK, bradykinin; A-II, angiotensin II; JA-2, N-[1(R,S)-carboxy-3-phenylpropyl]-Ala-Aib-Tyr-*p*-aminobenzoate; IPTG, isopropyl β-D-thiogalactoside; Abz, *o*-aminobenzoic acid; EDDnp, ethylenediamine-2,4-dinitrophenyl; E-64, *trans*-epoxysuccinyl-L-leucylamido-(4-guanido)butene; SNAP, S-nitroso-N-acetylpenicillamine; DTT, 1,4-dithiothreitol.

## Competing interests

The author(s) declare that they have no competing interests.

## Authors' contributions

TP designed and performed all biochemical and cell biological experiments, carried out data analysis and drafted and conceived the manuscript. AKC designed and helped in biochemical assays. EGR assisted in cell biological experiments, in vivo experiments and their design. VO designed and performed the mass spectrometry analysis and provided recombinant TOP and neurolysin. HPM assisted in the experiments of NO inhibition and SNAP assay. MAJ provided all FRET and free peptides, used as substrates. LJ provided the background knowledge on endo-oligopeptidases, including substrates and inhibitors. LRT, as Chairman of UNONEX, was the senior author who conceived the study, coordinated its execution, participated in its design and drafted and produced the final version of the manuscript. All authors read and approved the present version of the manuscript.
